# Comparison of porcine ALG and rabbit ATG on outcomes of HLA-haploidentical hematopoietic stem cell transplantation for patients with acquired aplastic anemia

**DOI:** 10.1186/s12935-021-02410-z

**Published:** 2022-02-21

**Authors:** Juan Chen, Yuanfeng Zhang, Xin Chen, Aiming Pang, Yuanqi Zhao, Li Liu, Runzhi Ma, Jialin Wei, Yi He, Donglin Yang, Rongli Zhang, Weihua Zhai, Qiaoling Ma, Erlie Jiang, Mingzhe Han, Jiaxi Zhou, Sizhou Feng

**Affiliations:** 1 State Key Laboratory of Experimental Hematology, National Clinical Research Center for Blood Diseases, Institute of Hematology and Blood Diseases Hospital, Chinese Academy of Medical Sciences and Peking Union Medical College, 288 Nanjing Road, Heping District, Tianjin, 300020 China; 2 Department of Hematology, The Affiliated Yantai Yuhuangding Hospital of Qingdao University, Yantai, 264000 Shandong Province China

**Keywords:** Anti-lymphocyte immunoglobulin, Anti-thymocyte immunoglobulin, Efficacy and safety, Acquired aplastic anemia, Haploidentical hematopoietic stem cell transplantation

## Abstract

**Objective:**

To evaluate the efficacy and safety of P-ALG (porcine anti-lymphocyte globulin) and R-ATG (rabbit anti-thymocyte globulin) in the conditioning regime for patients with acquired aplastic anemia who underwent HLA-haploidentical hematopoietic stem cell transplantation (halpo-HSCT).

**Methods:**

A total of 91 patients with acquired aplastic anemia who received haplo-HSCT at our center between January 2014 and December 2020 were retrospectively reviewed. Twenty-eight patients were in the P-ALG group while sixty-three patients were in the R-ATG group.

**Results:**

The median time was 11 versus 13 days (P = 0.294) for myeloid engraftment and 12.5 versus 15 days (P = 0.465) for platelet engraftment in the P-ALG and R-ATG groups, respectively. There were no significant difference in 5-year overall survival (74.83% ± 8.24% vs 72.29% ± 6.26%, P = 0.830), GVHD-free, failure-free survival (71.05% ± 8.65% vs 62.71% ± 6.22%, P = 0.662), failure-free survival (74.83% ± 8.24% vs 66.09% ± 5.84%, P = 0.647) and transplantation-related mortality (25.17% ± 8.24% vs 26.29% ± 6.22%, P = 0.708) between the two groups. The incidence of aGVHD (acute graft versus host disease) (65.39% ± 9.33% vs 62.71% ± 6.30%, P = 0.653), II–IV aGVHD (38.46% ± 9.54% vs 35.64% ± 6.24%, P = 0.695), III–IV aGVHD (19.23% ± 7.73% vs 10.53% ± 4.07%, P = 0.291), cGVHD (chronic graft versus host disease) (22.22% ± 12.25% vs 22.31% ± 6.30%, P = 0.915), and moderate to severe cGVHD (5.56% ± 5.40% vs 9.28% ± 4.46%, P = 0.993) were not significantly different. Similar outcomes were observed between the P-ALG and R-ATG groups for severe bacterial infection (17.9% vs 25.4%, P = 0.431), invasive fungal diseases (3.6% vs 9.5%, P = 0.577) and graft rejection (0% vs 9.5%, P = 0.218). However, the incidence of cytomegalovirus infection and Epstein-Barr virus infection was significantly lower in the P-ALG group (46.4% vs 71.4%, P = 0.022; 3.6% vs 25.4%, P = 0.014).

**Conclusion:**

The efficacy and safety of P-ALG were similar with R-ATG in the setting of haplo-HSCT for patients with acquired aplastic anemia patients. P-ALG could be an alternative for R-ATG.

**Supplementary Information:**

The online version contains supplementary material available at 10.1186/s12935-021-02410-z.

## Introduction

Acquired aplastic anemia (AA) is a rare bone marrow failure syndrome characterized by pancytopenia and hypocellular bone marrow which is clinically manifested by anemia, hemorrhage, and infection [1]. Currently, immunosuppressive therapy (IST) comprising antithymocyte globulin (ATG) with cyclosporine (CsA) and hematopoietic stem cell transplantation (HSCT) are the recommended treatment for severe AA/very severe AA (SAA/VSAA) and transfusion-dependent non-severe AA (NSAA). IST is the first line therapy for NSAA patients requiring transfusion support, SAA or VSAA patients without a matched sibling donor (MSD), and patients with older age [2]. For young patients (< 40 years), MSD HSCT should be the first-line therapy [3, 4]. However, only a small proportion of patients has a MSD, while almost every patient has a haploidentical donor. In recent years, more and more centers tried haplo-HSCT as the first-line therapy for young patients and demonstrated similar or better survival outcomes and acceptable complications compared with MSD-HSCT and IST [5–8]. ATG is a key drug in treatment of AA, whether in the IST or conditioning regime of HSCT. Rabbit ATG (R-ATG) and horse ATG (H-ATG) are widely used in different area of the world and proved to have good efficacy. However, horse ATG is not available in China, while a new porcine anti-lymphocyte globulin (P-ALG) is available. Several studies have compared the efficacy and safety of R-ATG and P-ALG in IST and suggested that P-ALG was similar to or even better than R-ATG [9–11]. In addition, P-ALG containing conditioning regime before HSCT in the setting of MSD-HSCT and MUD-HSCT have also demonstrated good efficacy and tolerance [12, 13]. However, P-ALG-containing conditioning regimen for haplo-HSCT has seldom been reported. Since haplo-HSCT has become an increasingly important treatment for AA patients, we aimed to evaluate the efficacy and safety of P-ALG comparing with R-ATG in the setting of haplo-HSCT in the paper.

## Patients and methods

### Patients and definitions

We retrospectively analyzed the data of 91 consecutive AA patients who received haplo-HSCT in stem cell transplantation center of the Institute of Hematology, Chinese Academy of Medical Science & Peking Union Medical College from January 2014 and December 2020. Institution and years for the patients who were treated with haplo-HSCT were listed in Additional file 1. For patients without events, the final date of follow-up was April 30, 2021. Of the 91 patients, 28 were categorized as SAA, 51 were VSAA and 12 were transfusion-dependent NSAA according to the diagnosis criteria [2]. Patients enrolled in the study did not have available MSD and voluntarily underwent haplo-HSCT. The exclusion criteria were as followings: patients with severe liver, kidney, heart, lung and other immunological diseases, patients who were pregnant, and whose bone marrow analysis were positive for myelodysplastic syndrome.

All patients and donors provided written informed consent for this protocol. For patients younger than 18 years old in the cohort, the consent was carried out by their patients. This study was approved by the Ethics Review Committee of the Institute of Hematology, Chinese Academy of Medical Science & Peking Union Medical College and was in compliance with the Declaration of Helsinki.

### Conditioning regime

All patients undergoing haplo-HSCT received conditioning based on Flu (fludarabine) 30 mg/m^2^/day × 5 days, Cy (cyclophosphamide) 50 mg/kg/day × 3 days, R-ATG (2.5 mg/kg/day × 5 days), or P-ALG (20–25 mg/kg/day × 5 days), with or without Bu (busulfan) 3.2 mg/kg/day × 2 days. Sixty-three patients received R-ATG, while twenty-eight patients received P-ALG as part of conditioning regime.

### GVHD prophylaxis

For GVHD prophylaxis, all transplant recipients received FK506 or CSA (cyclosporine A), short-term methotrexate, in addition to MMF (mycophenolate mofetil) or not.

### Supportive care

All patients resided in a class 100 laminar flow ward until neutrophil recovery. They routinely received antibiotic prophylaxis before transplantation: Compound Sulfamethoxazole Tablets 1.0 g twice a day for 1 week to prevent Pneumocystis carinii pneumonia and ganciclovir 10 mg/kg per day i.v. for 1 week to prevent CMV infection. For patients without history of invasive fungal disease (IFD) before transplantation, fluconazole was applied for prophylaxis of IFD until 3 months after transplantation. Patients who had IFD before transplantation received itraconazole, voriconazole, micafungin or caspofungin according to their pretransplant situations.

Patients received platelet transfusion when their platelet levels were below 20 × 10^9^/L or red blood cell transfusion if their hemoglobin levels were below 70 g/L. All patients received G-CSF (5 μg/kg once daily) from day + 6 until myeloid recovery.

CMV and EBV monitorization was done three times a week after transplantation when patients were in the hospital, and when they were out of hospital it was tested weekly until six months post transplantation.

### Criteria of outcomes

Engraftment was defined as ANC (absolute neutrophil counts) ≥ 0.5 × 10^9^/L for three consecutive days and platelet counts ≥ 20 × 10^9^/L without transfusion for 7 consecutive days.

The Mount Sinai Acute GVHD International Consortium (MAGIC) criteria was used to diagnose and grade acute GVHD (aGVHD) [14], while diagnosis and classification of chronic GVHD (cGVHD) was according to the 2014 National Institutes of Health consensus of cGVHD [15].

Graft rejection was defined as not reach the engraftment criterion of ANC ≥ 0.5 × 10^9^/L after transplantation or lose initial engraftment with minimal (< 5%) chimerism or entire recipient chimerism [16]. Chimerism status was evaluated by PCR of short tandem repeat sequences.

CMV (cytomegalovirus)—DNA was detected by plasma sample using real-time PCR and CMV viremia was defined as > 1000 copies/mL, and so was EBV (Epstein-Barr virus) viremia.

Invasive fungal disease (IFD) was defined according to the revised EORTC/MSG criteria [17]. Severe bacterial infection referred to bacteraemia or severe tissue infections. Regimen-related toxicity (RRT) was assessed according to Seattle Toxicity Criteria [18]. Overall survival (OS) was calculated from HSCT to death of any cause or last follow-up. GVHD-free, failure-free survival (GFFS), failure-free survival (FFS), transplantation-related mortality (TRM) were defined according to previous studies [6, 19]. GFFS (GVHD-free, failure-free survival) was defined as survival without grades III-IV acute GVHD, extensive chronic GVHD, and treatment failures. Treatment failures included death, relapse and primary or secondary graft failure. Failure-free survival (FFS) was defined as survival with response. Transplantation-related mortality (TRM) referred to death without relapse.

### Statistical analysis

The data were analyzed by the software GraphPad Prism 8, IBM SPSS statistics 25. The descriptive statistics for continuous variables and Chi-square test and Fisher’s exact test for categorical variables were used to compare incidence in univariate analysis. The Kaplan–Meier method was used to estimate the cumulative survival/incidence and differences were compared by the log-rank test. A two-sided P < 0.05 was considered as statistically significant.

## Results

### Characteristics of patients

A total of 91 patients were enrolled in the study. Twenty-eight and sixty-three patients were in the P-ALG group and R-ATG group, respectively. The baseline characteristics of patients in the two groups were listed in Table 1.

**Table 1:** Characteristics of patients

Characteristics	P-ALG (N = 28)	R-ATG (N = 63)	P value
Gender			
Male	13 (46.4%)	40 (63.5%)	0.128
Female	15 (53.6%)	23 (36.5%)	
Age (years)			
Median (range)	23 (6–55)	16 (4–52)	0.140
Diagnosis			
SAA	7 (25.0%)	21 (33.3%)	0.277
VSAA	15 (53.6%)	36 (57.1%)	
NSAA	6 (21.4%)	6 (9.6%)	
ATG before HSCT			
Yes	4 (14.3%)	9 (14.3%)	1.000
No	24 (85.7%)	54 (85.7%)	
Infection before transplantation			
Yes	3 (10.7%)	13 (20.6%)	0.396
No	25 (89.3%)	50 (79.4%)	
Interval from diagnosis to HSCT (months)			
Median (range)	4 (1–247)	3 (1–214)	0.248

### Transplantation details

Transplantation associated details including graft source, donor/recipient match (bloodtype, gender, HLA), GVHD prophylaxis and donor age between the two groups were similar (Table 2). The median dose of infused MNC and CD34^+^ cell in the P-ALG group were 10.66 × 10^8^/kg (range 5.08–22.68) and 3.44 × 10^6^/kg (range 2.02–8.60), which is not significantly different from the R-ATG group [MNC: 9.37 × 10^8^/kg (range 6.00–25.49) and CD34^+^ cell: 3.25 × 10^6^/kg (range 1.59–7.35)].

**Table 2:** Transplantation details

Conditioning regime	P-ALG (N = 28)	R-ATG (N = 63)	P value
	Flu, CY, P-ALG ± Bu	Flu, CY, R-ATG ± Bu	
Graft source			
PBSC	24 (85.7%)	47 (74.6%)	0.237
PBSC + BM	4 (14.3%)	16 (25.4%)	
Donor-recipient bloodtype match			
Matched	14 (50.0%)	34 (54.0%)	0.627
Major mismatched	7 (25.0%)	9 (14.3%)	
Minor mismatched	4 (14.3%)	13 (20.6%)	
Bidirectional mismatch	3 (10.7%)	7 (11.1%)	
Donor-recipient gender match			
Female to female	3 (10.7%)	9 (14.3%)	0.256
Female to male	5 (17.9%)	15 (23.8%)	
Male to female	12 (42.9%)	14 (22.2%)	
Male to male	8 (28.6%)	25 (39.7%)	
Donor-recipient HLA match			
5/10	15 (53.6%)	32 (50.8%)	0.108
6/10	3 (10.7%)	14 (22.2%)	
7/10	1 (3.6%)	10 (15.9%)	
8/10	4 (14.3%)	4 (6.3%)	
9/10	3 (10.7%)	2 (3.2%)	
10/10	2 (7.1%)	1 (1.6%)	
GVHD prophylaxis			
CSA + MTX	22 (78.6%)	45 (71.4%)	0.475
FK506 + MTX	6 (21.4%)	18 (28.6%)	
Donor-recipient CMV serostatus			
D^+^/R^+^	21 (95.5%)	54 (98.2%)	0.492
D^−^/R^+^	1 (4.5%)	1 (1.8%)	
D^+^/R^−^	0	0	
D^−^/R^−^	0	0	
Donor age (years)			
Median (range)	34 (9–54)	36 (8–62)	0.447
Dose of MNC (× 10^8^/kg)			
Median (range)	10.66 (5.08–22.68)	9.37(6.00–25.49)	0.411
Dose of CD34^+^ cells(× 10^6^/kg)			
Median (range)	3.44 (2.02–8.60)	3.25 (1.59–7.35)	0.827

### Engraftment

All patients except three patients (1 in the P-ALG group and 2 in the R-ATG group) with early death (< 14 days) had ANC engraftment, whereas 24 patients (85.7%) in the P-ALG group and 52 patients (82.5%) in the R-ATG group had platelet engraftment in 100 days post transplantation. The median time of ANC recovery in the P-ALG group and R-ATG group was 11 days (range 10–22) and 13 days (range 10–23), respectively. For platelet recovery, the median time was 12.5 days (range 8–95) and 15 days (range 10–83), respectively.

No patient in the P-ALG group experienced GR, while 6 patients (9.5%) developed GR in the R-ATG group.

### Infection

One patient in the P-ALG group and six patients in the R-ATG developed IFD (3.6% vs 9.5%, P = 0.577). Five patients in the P-ALG and sixteen patients in the R-ATG group developed severe bacterial infection (17.9% vs 25.4%, P = 0.431). The percentage of patients developing CMV viremia and EBV viremia in the P-ALG group were 46.4% (13/28) and 3.6% (1/28), respectively. While in the R-ATG group, the incidence of CMV viremia and EBV viremia was 71.4% (45/63) and 25.4% (16/63), respectively. The incidence of CMV viremia and EBV viremia was significantly lower in the P-ALG group than the R-ATG group (P = 0.022 and P = 0.014, respectively).

### GVHD

In the P-ALG group, 17 patients developed aGVHD, and 5 of whom were grade III-IV aGVHD. In the R-ATG group, 37 patients developed aGVHD, and 6 of whom were grade III-IV aGVHD. There was no significant difference of aGVHD between the two groups. The incidence of cGVHD or moderate to severe cGVHD of P-ALG group and R-ATG group were also similar (22.22% ± 12.25% vs 22.31% ± 6.30%, P = 0.915, 5.56% ± 5.40% vs 9.28% ± 4.46%, P = 0.993, respectively) (Table 3, Fig. 2).

**Table 3:** Outcomes of patients

Outcomes	P-ALG (N = 28)	R-ATG (N = 63)	P value
Time of engraftment			
ANC, days (range)	11 (10–22)	13 (10–23)	0.294
PLT, days (range)	12.5 (8–95)	15 (10–83)	0.465
Infection			
CMV			
Yes	13 (46.4%)	45 (71.4%)	0.022
No	15 (53.6%)	18 (28.6%)	
EBV			
Yes	1 (3.6%)	16 (25.4%)	0.014
No	27 (96.4%)	47 (74.6%)	
IFD			
Yes	1 (3.6%)	6 (9.5%)	0.577
No	27 (96.4%)	57 (90.5%)	
Severe bacterial infection			
Yes	5 (17.9%)	16 (25.4%)	0.431
No	23 (82.1%)	47 (74.6%)	
Graft rejection			
Yes	0 (0%)	6 (9.5%)	0.218
No	28 (100%)	57 (90.5%)	
GVHD			
aGVHD, %	65.39 ± 9.33	62.71 ± 6.30	0.653
II–IV aGVHD, %	38.46 ± 9.54	35.64 ± 6.24	0.695
III–IV aGVHD, %	19.23 ± 7.73	10.53 ± 4.07	0.291
cGVHD, %	22.22 ± 12.25	22.31 ± 6.30	0.915
Moderate-severe cGVHD, %	5.56 ± 5.40	9.28 ± 4.46	0.993
5-year OS, %	74.83 ± 8.24	72.29 ± 6.26	0.830
5-year GFFS, %	71.05 ± 8.65	62.71 ± 6.22	0.662
5-year FFS, %	74.83 ± 8.24	66.09 ± 5.84	0.647
5-year TRM, %	25.17 ± 8.24	26.29 ± 6.22	0.708

### Deaths and survival

**Fig. 1:**
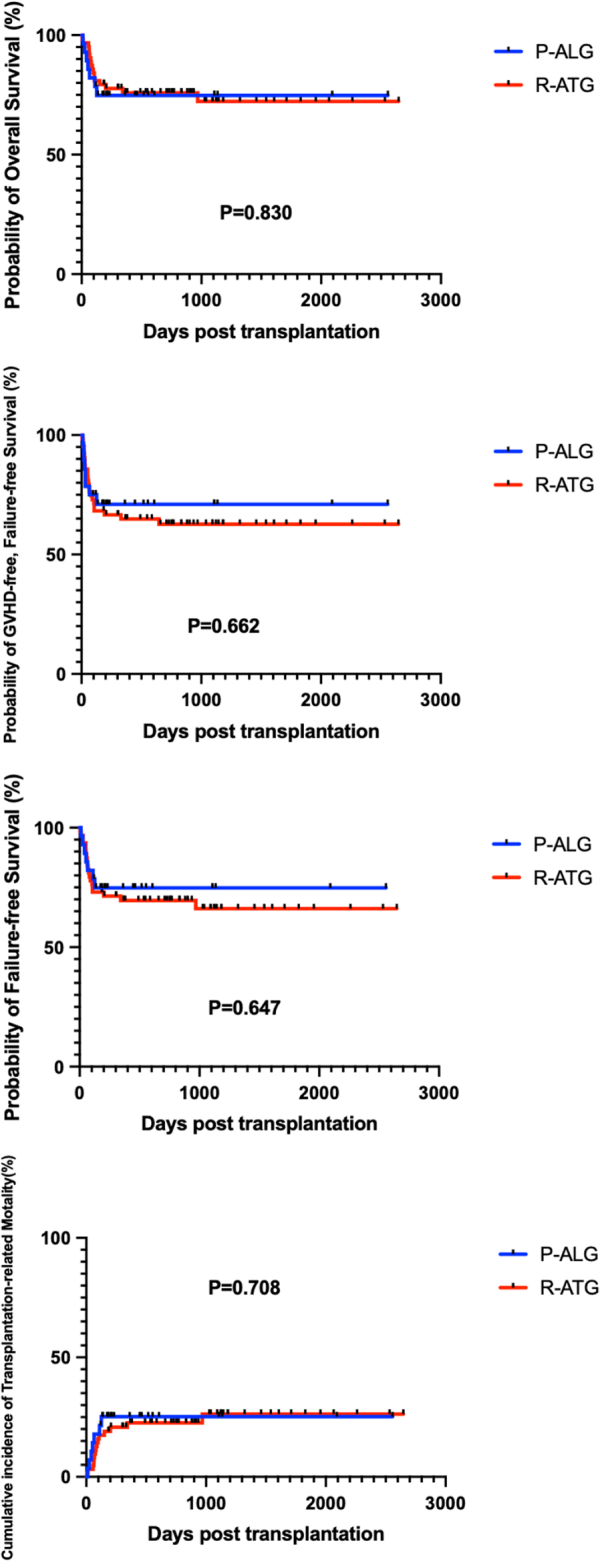
Survival curves

**Fig. 2:**
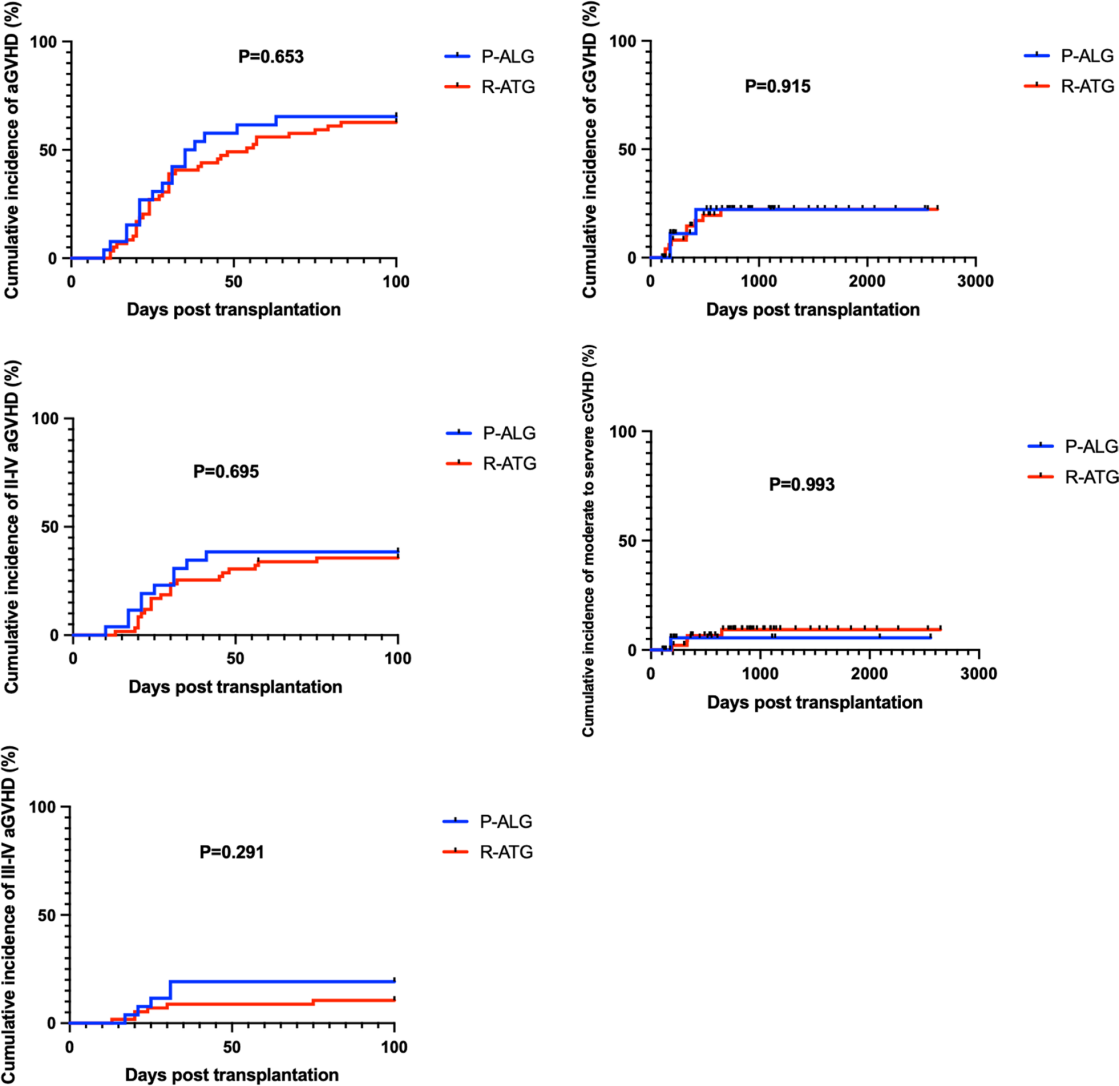
Incidence of aGVHD and cGVHD

Seven patients died in the P-ALG group, 5 with aGVHD and 2 with infection. Sixteen patients died in the R-ATG group, 4 with aGVHD, 3 with cGVHD, 6 with infection, 2 with graft rejection, and 1 with intracranial hemorrhage, respectively. In the P-ALG group, at a median follow-up of 212 days (range from 10 to 2557), 21 patients survived and the 5-year OS was 74.83% ± 8.24%. In the R-ATG group, at a median follow up of 729 days (range from 6 to 2648), 47 patients survived and the 5-year OS was 72.29% ± 6.26%. There was no significant difference in terms of the 5-year OS between the 2 groups (P = 0.830), and so were the 5-year GFFS, 5-year FFS and 5-year TRM (Table 3, Figs. 1, 2).

## Discussion

Immunosuppressive therapy and MSD-HSCT are the frontline therapy for AA patients. Since a matched sibling donor is not available for every patient, haplo-HSCT has increasingly become a therapeutic option for patients with AA due to optimal conditioning regimens and improved supportive care. In recent years, a series of studies [5–8, 20] suggested that haplo-HSCT showed an overall efficacy comparable to those with IST and MSD-HSCT.

Horse or rabbit ATG is commonly used worldwide, while horse ATG is not available in China. Porcine ALG is a product developed in China and was approved by the Sino Food and Drug Administration in 2004. A series of retrospective studies evaluating the efficacy of P-ALG in treatment of AA were conducted [8–13, 21–24]. P-ALG was reported to have similar or superior overall response at 6 months compared to R-ATG (64.0–79.4% vs 48.1–64.7%) in the IST treatment for AA patients [25]. Liu et al. [21] retrospectively analyzed SAA patients treated with either P-ALG (n = 43) or R-ATG (n = 32) plus CSA, and suggested there were no significant difference in 2-year OS between the 2 groups (87.4% ± 6.2% vs 83.2% ± 7.8%, P = 0.493). Other studies [10, 11] comparing the efficacy of P-ALG and R-ATG in the IST treatment showed similar results.

Moreover, some studies have compared the efficacy of P-ALG and R-ATG in the conditioning regime before transplantation. We have previously compared the efficacy of P-ALG and R-ATG in MSD-HSCT for patients with SAA, including 55 patients in the P-ALG group and 58 patients in the R-ATG group [12]. There was also no significant difference in 3-year overall survival (84.4% ± 5.7% in the P-ALG group vs 93.1% ± 3.3% in the R-ATG group, P = 0.235), whereas the incidence of aGVHD and cGVHD was higher in P-ALG group (20.7% ± 5.3% vs 43.4% ± 7.0%, P = 0.015; 20.1% ± 5.8% vs 46.0% ± 7.9%, P = 0.003). The higher incidence of aGVHD and cGVHD might be related to older age and more PBSC as graft source in the P-ALG group. Recently, Li et al. [13] evaluated the outcomes of 41 SAA patients receiving a P-ALG-containing conditioning regimen before MSD-HSCT and matched-unrelated HSCT (URD-HSCT). The actuarial 3-year OS and disease-free survival (DFS) were 95.1% ± 3.4% and 85.0% ± 5.7%, respectively. The cumulative incidence of grades III to IV aGVHD and 5-year cumulative incidence of moderate-severe cGVHD was 4.9% ± 3.4% and 10.8% ± 5.1% , respectively. These studies suggested the P-ALG-containing regimen has satisfactory effects and safety in MSD-HSCT and URD-HSCT for SAA patients. However, the efficacy and safety of P-ALG in haplo-HSCT has seldom been reported.

In this study, we wanted to compare the efficacy and safety of P-ALG and R-ATG in the setting of haplo-HSCT. The baseline characteristics and transplantation details of patients of the two groups were similar (Tables 1, 2).

The incidence of aGVHD, II-IV aGVHD and III-IV aGVHD in the P-ALG group and R-ATG group were comparable and so were the incidence of cGVHD and moderate to severe cGVHD (Table 3). And the incidence rate of aGVHD and cGVHD were similar to previous reported in the setting of haplo-HSCT [6].

There were no significant difference of IFD (P = 0.577) and severe bacterial infection (P = 0.431), graft rejection (P = 0.218) and engraft time (Table 3).

Interestingly, in our study, the incidence of CMV viremia and EBV viremia was significantly lower in the P-ALG group than the R-ATG group (46.4% vs 71.4%, P = 0.022 and 3.6% vs 25.4%, P = 0.014). Previous studies of ATG/ALG in IST indicated more clearance of peripheral blood lymphocytes by R-ATG than by P-ALG [9, 10, 25]. Patients in R-ATG group had a significantly lower minimum number of lymphocytes than the P-ALG group and remained significantly lower after 1, 3 and 6 months of treatment initiation and recovered to equivalent levels after 12 months [10]. Furtherly, when comparing the changes of subsets in T cells, the inhibitory effects on CD4^+^ T cells were significantly higher in the R-ATG group than in the P-ALG group, while the inhibitory effects on the CD8^+^ T cells were not different between the two groups. Thus, the stronger immunosuppressive effects of R-ATG than P-ALG, mainly on CD4^+^ T cells, may possibly account for the higher incidence of CMV and EBV infection in R-ATG group than the P-ALG group.

Until the date of last follow-up, 21 patients and 47 patients were alive in the P-ALG group and R-ATG group, respectively. The 5-year OS, GFFS and FFS and TRM were similar in the two groups (P = 0.830, P = 0.662, P = 0.647, P = 0.708).

In conclusion, P-ALG achieved similar survival and safety profiles compared with R-ATG. The present study provided evidence in clinical application of P-ALG in the conditioning regime of haplo-HSCT. P-ALG could be a potential alternative preparation for R-ATG. However, due to the retrospective origin and small sample size, further prospective, large-scaled clinical trials are needed to investigate the effects and explore the underlying molecular mechanisms for ATG/ALG.

## Supplementary Information

**Additional file 1.** Institution and years for the patients who were treated with haplo-HSCT

## Data Availability

The data and materials can be obtained from the first author and corresponding author.
